# Comparing Different Data Partitioning Strategies for Segmenting Areas Affected by COVID-19 in CT Scans

**DOI:** 10.3390/diagnostics14242791

**Published:** 2024-12-12

**Authors:** Anne de Souza Oliveira, Marly Guimarães Fernandes Costa, João Pedro Guimarães Fernandes Costa, Cícero Ferreira Fernandes Costa Filho

**Affiliations:** 1 R&D Center in Electronic and Information Technology, Federal University of Amazonas, Manaus 69077-000, Brazil; anneolllivveira@gmail.com (A.d.S.O.); mcosta@ufam.edu.br (M.G.F.C.); 2 Cancer Institute of São Paulo State, São Paulo 01246-000, Brazil; joaopedrogfc@gmail.com

**Keywords:** computed tomography, convolutional neural networks, coronavirus, COVID-19, deep learning, image segmentation, lung, radiology

## Abstract

Background/Objectives: According to the World Health Organization, the gold standard for diagnosing COVID-19 is the Reverse Transcription Polymerase Chain Reaction (RT-PCR) test. However, to confirm the diagnosis in patients who have negative results but still show symptoms, imaging tests, especially computed tomography (CT), are used. In this study, using convolutional neural networks, we compared the following topics using manual and automatic lung segmentation methods: (1) the performance of an automatic segmentation of COVID-19 areas using two strategies for data partitioning, CT scans, and slice strategies; (2) the performance of an automatic segmentation method of COVID-19 when there was interobserver agreement between two groups of radiologists; and (3) the performance of the area affected by COVID-19. Methods: Two datasets and two deep neural network architectures are used to evaluate the automatic segmentation of lungs and COVID-19 areas. The performance of the U-Net architecture is compared with the performance of a new architecture proposed by the research group. Results: With automatic lung segmentation, the Dice metrics for the segmentation of the COVID-19 area were 73.01 ± 9.47% and 84.66 ± 5.41% for the CT-scan strategy and slice strategy, respectively. With manual lung segmentation, the Dice metrics for the automatic segmentation of COVID-19 were 74.47 ± 9.94% and 85.35 ± 5.41% for the CT-scan and the slice strategy, respectively. Conclusions: The main conclusions were as follows: COVID-19 segmentation was slightly better for the slice strategy than for the CT-scan strategy; a comparison of the performance of the automatic COVID-19 segmentation and the interobserver agreement, in a group of 7 CT scans, revealed that there was no statistically significant difference between any metric.

## 1. Introduction

In the previous decades, none of the coronaviruses had caused serious illness in humans, but on 31 December 2019, the World Health Organization was alerted to cases of pneumonia in the city of Wuhan, China. These were confirmed on 7 January 2020 to belonging to a new coronavirus variant, SARS-CoV-2. The infectious disease caused by the SARS-CoV-2 coronavirus has been named COVID-19 [1].

On 11 March 2020, COVID-19 was characterized as a pandemic due to the geographical distribution of the disease in several countries around the world. The severity of COVID-19 was defined by the World Health Organization as critical, severe, and not severe [2] and the symptoms listed were a fever, tiredness, and a dry cough, as well as less common symptoms such as a loss of taste or smell, nasal congestion, conjunctivitis, sore throat, headache, muscle or joint pain, skin rashes, nausea or vomiting, diarrhea, chills, and dizziness.

The diagnostic confirmation of COVID-19 is realized with the identification of viral RNA via a Reverse Transcription Polymerase Chain Reaction (RT-PCR) test, which is considered the gold-standard test. However, confirmation of the diagnosis can be performed via chest imaging in situations where there are symptoms of the disease, but RT-PCR is not available; there is a delay in obtaining the results; or the RT-PCR results are initially negative, but there are symptoms that suggest the disease.

Chest imaging tests include chest radiography, chest computed tomography (CT), and lung ultrasound analysis. In a systematic review carried out in [3], for the first months of the COVID-19 pandemic, 23 studies were found that used these three types of imaging to assess the accuracy of the diagnosis in relation to a reference standard. Overall, 3 of these used chest X-ray exams, 19 used chest CT scans, and 1 used a lung ultrasound. Among the 19 CT studies, 5 used artificial intelligence (AI) to interpret the images. Among the studies that used AI, two used deep learning models, with one applying convolutional neural networks (CNNs). The tasks performed in these studies largely involved classification, where the model determines whether the CT classified the patient as with or without COVID-19.

In the literature review carried out in this work, studies were also found that used deep learning models trained with convolutional neural networks to delimit the regions affected by COVID-19 in CT scans. Most of the articles used to segment areas affected by COVID-19 only use the features extracted by the deep network itself. It should be noted, however, that some approaches use the fusion of these features with handcrafted features extracted using the Weber Local Descriptor (WLD). The authors in [4] claim that, regarding fusion characteristics, the results are better than those obtained with only ResNet101 or DensNet201.

To undertake this review, the databases selected were PubMed, IEEE, ScienceDirect, and ACM and the general expression used in the query was as follows: ((machine learning) AND (deep learning)) AND ((image segmentation) AND (computerized tomography)) AND ((lung infection) AND (coronavirus OR COVID-19)). We made appropriate adaptations for each database. The total number of articles returned without repetition was 56.

In the screening phase, 31 articles were retained and 25 were excluded according to the following established criteria: 1. The article was included if it trained convolutional neural networks to segment COVID-19. 2. The article was included if it cited the databases used. 3. An article was included if it cited the data partitioning used for training and testing (CT-scan or slice strategy). 4. The article was included if it cited the networks used to perform the segmentation task(s). 5. The article was included if it cited the Dice metric.

In the analysis phase, 22 articles were retained and 9 were excluded. The criteria established were as follows: 1. the article was included if it cited the network architecture used to segment the lungs, COVID-19, or both; 2. the article was included if it cited the type of method adopted, whether automatic or iterative; and 3. the article was included if it aimed to train a model to segment COVID-19.

After analyzing the articles, we observed that segmenting the affected areas is of fundamental importance for the clinical follow-up of patients, as it makes it possible to monitor the proportion of COVID-19 in the lung region on CT scans, and that studies dealing with COVID-19 segmentation can be classified according to the number of steps used in the segmentation process and according to the methodology used for data partitioning.

With respect to the number of steps used in the segmentation process, we identified two approaches in the literature. First, as shown in Table 1, COVID-19 segmentation is carried out in a single step [5,6,7,8,9,10,11,12,13,14,15,16,17,18,19]. This approach is referred to in this article as one-step segmentation. In the second approach, as shown in Table 2, the segmentation of the area affected by COVID-19 is preceded by a lung segmentation step [20,21,22,23,24,25,26]. This approach is referred to in this article as two-step segmentation.

With respect to the methodology used for data partitioning (DP), we also identified two strategies. In the first strategy, the data are divided by CT scans, and the slices of a patient’s CT scan are only used for training or testing the classifier, as shown in [5,6,7,8,9,10,11,20,21,22]. In the second strategy, the data are divided into slices. In this strategy, slices from the same patient’s CT scan can be used for both training and testing the classifier, as shown in [12,13,14,15,16,17,18,19,23,24,25,26]. This first strategy is referred to in this article as the CT-scan strategy, and the second strategy is referred to as the slice strategy.

Table 1 (one-step segmentation methods) shows the papers with the highest values in terms of the Dice metric. Importantly, among these studies, refs. [5,6] adopted the CT-scan strategy for data partitioning, which presented Dice metric values of 92.75% and 91.60%, respectively, and used interactive methods to obtain COVID-19 segmentation models. The other studies used automatic methods to segment COVID-19, with [7] achieving a Dice metric of 80.29%. Among the studies that adopted the slice strategy for data partitioning, all used automatic methods, with the highest value for the Dice metric achieved in the work of [11], at 92%, followed by the work of [13], at 88%. The studies in [5,14,15,16] employed one of the datasets used in this work: the COVID-19 CT Lung and Infection Segmentation Dataset [27]. This dataset is referred to in this work as COVID-19 CT Seg.

All the papers in Table 2 (two-step segmentation methods) implemented automatic segmentation methods. Among the studies that adopted the CT-scan strategy for data partitioning, we highlight those of [20,21,22], which obtained Dice metric values of 71.30%, 67.30%, and 63.20%, respectively. Among the studies that adopted a slice strategy for data partitioning, we highlight those of [23,24,25,26], with Dice metrics of 85.00%, 79.80%, 78.06%, and 74.90%, respectively.

Some studies [21,22,24,25] employed the datasets used in this work: COVID-19 CT Seg [27] and/or MedSeg COVID Dataset 2 [28]. The latter is referred to in this work as MedSeg. Ref. [21] used nnU-Net to segment the left and right lungs and detect COVID-19 infection. In [22], the U-Net architecture was used to segment regions infected with COVID-19. Ref. [24] proposed the Anam-Net architecture and provided the percentages of normal and COVID-19-infected regions. In [25], the RCNN-ResNeSt-200 mask-cascade architecture was used in the first module of the three proposed by the authors to train and evaluate lung and COVID-19 segmentation models.

Among the studies listed in Table 1 and Table 2, we observed that no study compared the automatic segmentation of COVID-19 using two DP strategies, i.e., CT-scan and slice methods; that one study presented a comparison of the performance of the automatic segmentation of COVID-19 with the performance of the segmentation conducted by two groups of radiologists on the same set of images and the interobserver agreement between these two groups [8]; and that two studies used interactive methods to segment COVID-19, i.e., they relied on human intervention in the segmentation process [5,6].

In view of what has been described above, the contributions of this work are as follows:

- Using CT images, we compare the performance of deep learning tools by employing the two-step segmentation method for COVID-19; we use both the data partitioning strategies, i.e., CT-scan and slice methods.
- Using CT images, we compare the performance of deep learning tools by employing a two-step segmentation method for COVID-19, with the agreement level of segmentation assessed by two distinct groups of radiologists for the same dataset.
- Using CT images, we compare the performance of deep learning tools in a two-step segmentation process for COVID-19 employing manual and automatic lung segmentation.

## 2. Materials and Methods

### 2.1. Materials

**Figure 1:**
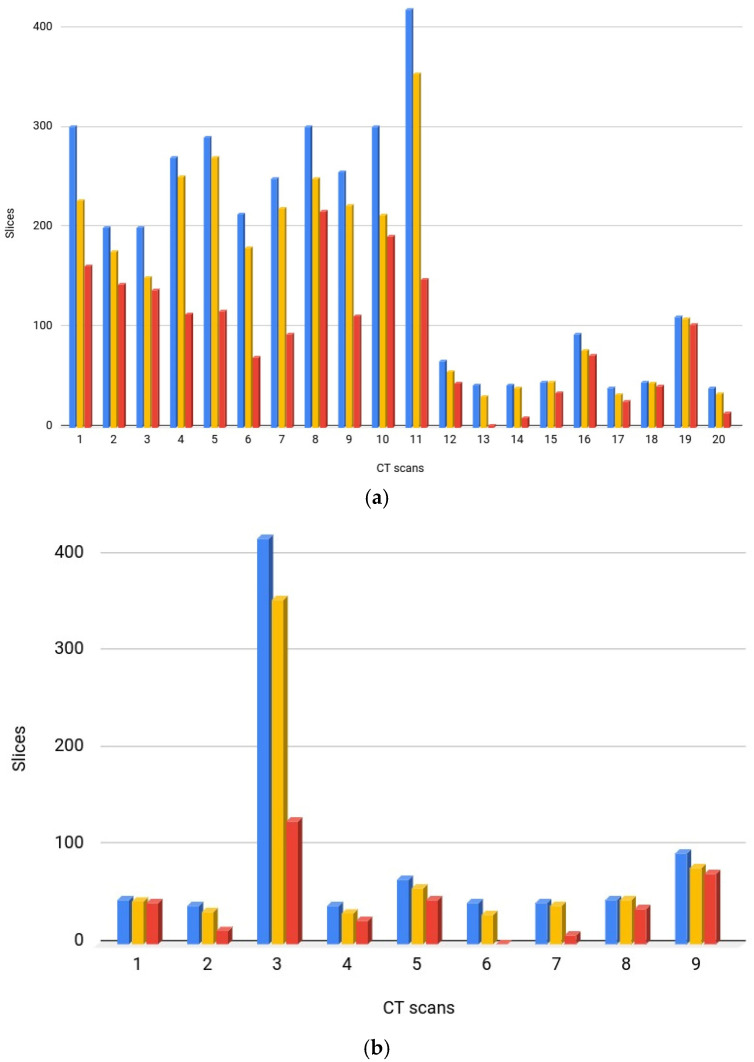
Graphs showing, for each CT scan of the dataset, the total number of slices, the number of slices containing the lung, and the number of slices containing regions with COVID-19. (**a**) Dataset 1; (**b**) Dataset 2. Blue bars: number of slices; yellow bars: number of slices with lung; red bars: number of slices with COVID-19.

Dataset 1, known as COVID-19 CT Seg, consists of 20 CT scans [27]. This dataset contains 3520 slices that were assessed in 2 stages, an initial assessment and a review, by 2 radiologists. A total of 1644 slices were positive. Figure 1a shows the number of slices per CT scan in this dataset. CT scans, lung masks, and COVID-19 masks were available. The number of slices with COVID-19 in each CT scan ranged from 2 to 216. In the CT scans, the patient’s chests were turned to the right and left. The original sizes of the images were 630 × 630 pixels and 512 × 512 pixels. All image regions associated with COVID-19 were labeled with ground glass opacities.

Dataset 2, known as MedSeg, consists of 9 CT scans [28]. This dataset has 829 slices, of which 373 slices are positive, that were evaluated by a radiologist. Figure 1b shows the number of slices per CT scan in this dataset. It contains 7 CT scans in common with Dataset 1. As the COVID-19 areas of the exams in Dataset 2 were evaluated by a different group of radiologists, it was possible to compare the level of agreement of the method proposed in this study with the results of the two groups of radiologists when segmenting the COVID-19 area in the 7 CT scans in common.

### 2.2. Methods

**Figure 2:**
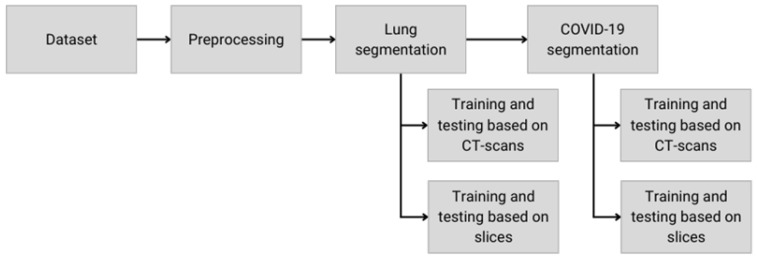
The steps of the methodology adopted in this work.

This work uses two-step segmentation. Figure 2 illustrates the methodology adopted to evaluate COVID-19 area segmentation in CT scans. Two ways of dividing the dataset are evaluated, namely, the slice and the CT-scan strategies.

#### 2.2.1. Preprocessing

**Figure 3:**
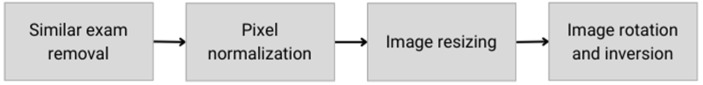
Steps used in image preprocessing.

The preprocessing steps are shown in Figure 3. In the first step, a radiologist of our research group evaluated all the CT scans of both datasets and reported that two CT scans from Dataset 1 were identical, with the only difference being that one of them contained more slices than the other. Therefore, one of the exams was removed and Dataset 1 was left with 19 CT scans.

The pixels of each slice, in both datasets, were normalized to the range 0–255 according to the MAX-MIN normalization process shown in Equation (1) [29]:

(1) $$f_{n}=\frac{f_{0}-f_{min}}{f_{max}-f_{min}}$$

where

f_n_—normalized pixel value;
f_0_—original pixel value;
f_min_—minimum pixel value in the slice;
f_max_—maximum pixel value in the slice.

**Figure 4:**
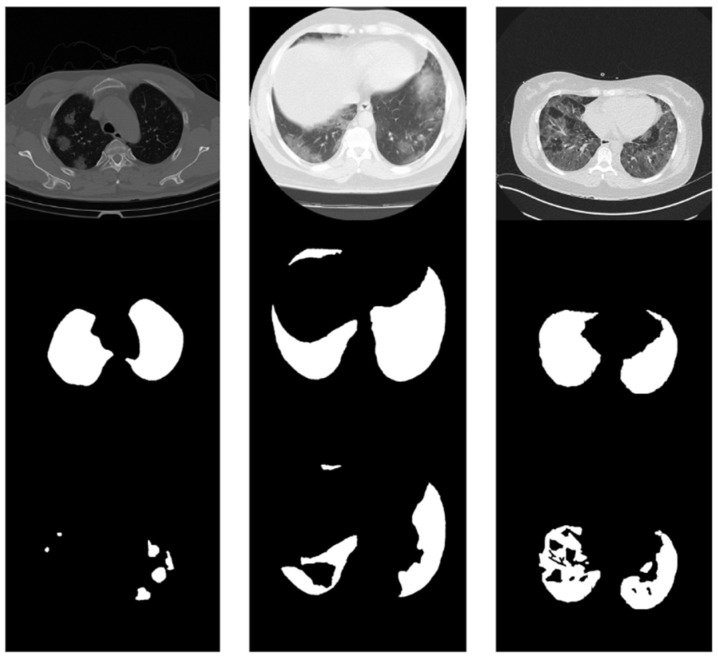
Examples of slices of CT scans after preprocessing. From top to bottom, a slice, a lung mask, and a COVID-19 mask.

All the images were resized to 512 × 512 pixels. In this step, one CT scan of Dataset 1, which presented a different ratio between height and width, was removed. Therefore, Dataset 1 was left with only 18 CT scans. To ensure all the CT scans to had their chests facing down and text annotations in the correct position, rotation and inversion operations were applied in the last preprocessing step. Figure 4 shows examples of CT-scan slices after all modifications were performed.

#### 2.2.2. DP Strategies for Training, Validation and Testing

**Figure 5:**
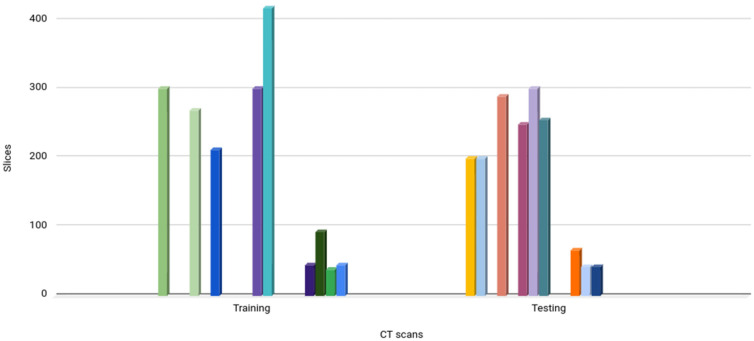
Training and test sets obtained with the CT-scan strategy. Different colors represent different CT-scans.

For classifier evaluation, two DP strategies were used to divide the datasets into training and testing sets. The first one was the CT-scan strategy, which uses different CT scans for training and testing a classifier. This strategy is also known as the independent subject strategy because the CT scans of a patient are only used for training or for testing, not for both. A random process was used to select the CT scans from both sets. Figure 5 shows the results of the CT-scan strategy.

**Figure 6:**
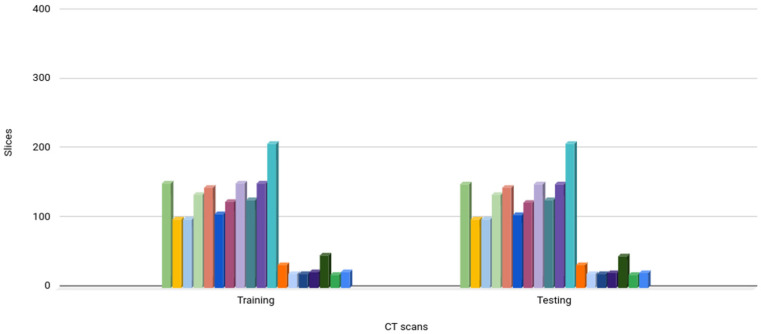
Training and test sets obtained with the slice strategy. Different colors represent different CT-scans.

The second method was the slice strategy, which uses different slices of the same CT scan for both training and testing a classifier. This strategy is also known as the dependent subject strategy because slices from a CT scan of a patient are used for training and testing. An interleaved process was used to select the slices in each CT scan. From a given CT scan, 50% of the slices were used for training and 50% were used for testing. Figure 6 shows the results of the slice strategy.

All training sets were split at runtime to create a validation set. This procedure was carried out during the model training phase and a 75:15 ratio was adopted.

A survey of the images showed that the area affected by COVID-19 in the CT scans varied from 0.01% to 29.57%, which guaranteed a good diversity of images.

#### 2.2.3. Classifiers

**Figure 7:**
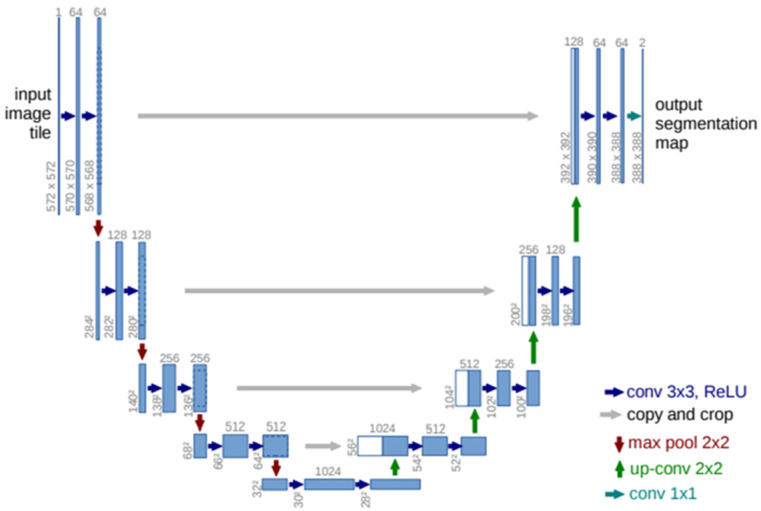
Convolutional neural network architecture used for semantic segmentation of lungs and COVID-19—U-Net [29].

Two convolutional neural networks were used in this work for semantic segmentation. The first architecture, shown in Figure 7, was U-Net [30]. The authors evaluated this architecture for use in the segmentation of neuronal structures in electron microscopic recordings and cell segmentation in light microscopic images using 2 different datasets and achieved good performances for all the evaluated tasks. U-Net consists of an encoder with 4 subsampling stages. Each subsampling stage is composed of 2 convolution layers with a 3 × 3 kernel followed by a 2 × 2 maxpooling layer. The decoder consists of 4 oversampling stages. Each oversampling stage is composed of a 2 × 2 upconvolution layer, a concatenation with the corresponding feature map from the encoder, and 2 convolution layers with 3 × 3 kernels. This architecture connects encoder layers with decoder layers. U-Net is widely used in semantic segmentation tasks, particularly in medical image processing. It is used to perform accurate segmentation when there are few training images. In some of the experiments in our work, to improve generalization, a dropout layer was inserted between the Conv2D layer with 1024 filters and the Conv2DTranspose layer with 512 filters.

**Figure 8:**
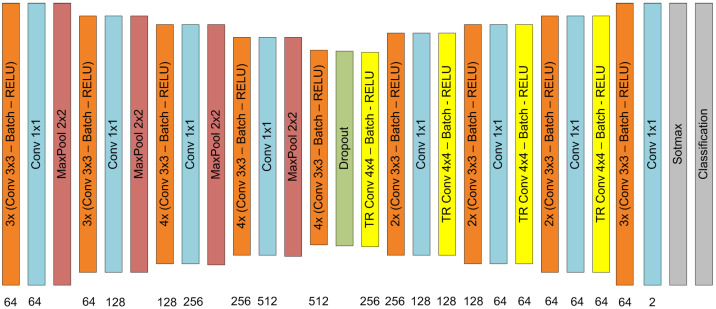
Convolutional neural network architecture used for semantic segmentation of COVID-19—CNN2.

The second architecture, CNN2, is shown in Figure 8 and was proposed by our research group [31] in order to segment the lumen in intravascular optical coherence tomography images. Like U-Net, this architecture can also be divided into two parts: downsampling and upsampling sections. The downsampling section is composed of blocks of the convolution layer–batch layer–ReLU layer. The convolution layers have filters of size 3 × 3 and padding 1. These blocks are followed by a convolution layer with a filter size of 1 × 1 and a MaxPool layer with a filter size of 2 and stride of 2. At the downsampling end, there is a dropout layer. At the architecture output, there is a Softmax layer and a classification layer. At the upsampling section, between the transposed convolution layers, there is a block composed of a convolution layer with a filter size of 3 × 3–batch layer–ReLU layer–convolution layer with a filter size of 1 × 1. At the output, there is a Softmax layer and a classification layer.

The optimal hyperparameters chosen, after some experiments, to train both architectures are shown in Table 3. During the training phase, we randomly evaluated some options for the following hyperparameters: optimizer (RMSProp, ADAM and gradient descent with momentum); batch size (8, 16, 32); initial learning rate (10^−3^, 10^−4^); dropout rate (0.4, 0.5 and 0.6); L_2_ regularization (10^−2^, 10^−3^, 10^−4^).

Deep learning models, with their complex architectures and numerous parameters, are susceptible to overfitting, especially when trained on small or imbalanced datasets. This occurs when the model learns the training data too well, leading to poor generalization of unseen data. Techniques like regularization, dropout, data augmentation, and early stop can mitigate this issue, but it remains a significant challenge. In this work, we used these four techniques to mitigate the overfitting. The regularization method used was the L_2_ technique. The data augmentation consisted of applying 10 rotations of 36^0^ to the original images of the training sets. The early stop had a patience of 3.

#### 2.2.4. Lung and COVID-19 Segmentation

For lung segmentation, only the U-Net architecture was employed. For each DP strategy, 8 experiments were performed, combining dropout (D), L_2_ regularization (L), and data augmentation (A). For COVID-19 segmentation, both U-Net and CNN2 architectures were employed. For each strategy, 8 experiments were also performed using the same combination mentioned. COVID-19 segmentation was performed via both automatic and manual lung segmentation. For manual lung segmentation, the radiologists’ lung labels were used.

### 2.3. Simulation Environment and Evaluation Metrics

The environment used to write the codes for training and testing the models was a cloud service hosted by Google, namely, Google Colab. The software used to implement the codes was Keras, a high-level deep learning API written in Python. The machine learning kernel used with Keras was TensorFlow 2.12.0, an open-source platform.

The version of Python installed on the cloud computer was 3.9.16. All the generated codes and models were stored on Google Drive. To expand Google Drive’s storage capacity, Google One was added to the environment. This work adopted a public cloud architecture with private resources. The configuration of the public cloud architecture was as follows: storage capacity, 100 GB; RAM memory, 35 GB; processor, Intel(R) Xeon(R) CPU @ 2.30 GHz; operational system, Linux with Ubuntu 20.04.3 LTS; tensor processor unit, TPU v2; and RAM memory of the tensor unit, 16 GB. To gain access to continuous code execution, increase the amount of RAM, and access the TPU, Google Colab Pro was added. The TPU consists of four TPU chips and 16 GB of high-bandwidth memory (HBM).

To evaluate the model performances, the following metrics were employed: accuracy—Equation (2); global accuracy—Equation (3); Dice—Equation (4); and Jaccard—Equation (5).

(2) $$Accuracy=\frac{\frac{TP}{TP+FN}+\frac{TN}{TN+FP}}{2}$$

(3) $$Globalaccuracy=\frac{TP+TN}{TP+TN+FP+FN}$$

(4) $$Dice=\frac{2TP}{2TP+FP+FN}$$

(5) $$Jaccard=\frac{TP}{TP+FP+FN}$$

where:

TP—true positives;
TN—true negatives;
FP—false positives;
FN—false negatives.

To assess whether the differences between the mean values of two metric values were statistically significant, Student’s *t*-test was used, with a significance level of 1%. To assess whether the Student’s *t*-test could be applied to the results, the normality of the data was evaluated using the Shapiro–Wilk test. When the normality condition was not met, the Mann–Whitney test was applied.

## 3. Results

In this section, we present results concerning the following topics: lung segmentation, COVID-19 area segmentation via automatic lung segmentation, COVID-19 area segmentation via manual lung segmentation, and a comparison between automatic COVID-19 segmentation and interobserver agreement.

### 3.1. Lung Segmentation Results

For both DP strategies, better results were obtained with U-Net + D + L (CT-scan strategy, Dice = 99.79%; slice strategy, Dice = 98.74%). Table 4 shows the results for lung segmentation in the test set when the best validation models were used.

As shown in Table 4, the results obtained with the slice strategy are better than the results obtained with the CT-scan strategy. Nevertheless, there are no statistically significant differences. For accuracy, the t-value = 2.17, *p* > 0.01 (*p* = 0.019797), 95% CI: CT scan—[96.293–98.907], and Slice—[98.395–99.125]. For global accuracy, the t-value = 2.07, *p* > 0.01 (*p* = 0.024445), 95% CI: CT scan—[98.135–99.885], and Slice—[99.572–99.748]. For Dice, the t-value = 2.42, *p* > 0.01 (*p* = 0.011382), 95% CI: CT scan—[91.886–98.694], and Slice—[97.907–98.673]. For Jaccard, the t-value = 2.44, *p* > 0.01 (*p* = 0.010892), 95% CI: CT scan—[91.805–98.495], and Slice—[97.719–98.541]. To ensure the validity of the Student’s *t*-test, we applied the Shapiro–Wilk test to the accuracies, global accuracies, and Dice and Jaccard values, and obtained W(27) = 0.77, *p* < 0.01 (*p* = 4.38 × 10^−5^); W(27) = 0.44, *p* < 0.01 (*p* = 4.12 × 10^−9^); W(27) = 0.53, *p* < 0.01 (*p* = 3.83 × 10^−8^); and W(27) = 0.55, *p* < 0.01 (*p* = 5.70 × 10^−8^), respectively.

### 3.2. COVID-19 Segmentation Results

#### 3.2.1. COVID-19 Segmentation Results with Automatic Lung Segmentation

For the U-NET network, the best results for the CT-scan and slice strategies were obtained with U-NET (Dice = 87.30%) and U-NET + A + D (Dice = 86.61%), respectively. For CNN2, the best results for the CT-scan and slice strategies were obtained with CNN2 + D (Dice = 87.30%) and CNN2 + A (Dice = 86.61%), respectively.

Table 5 shows the results obtained in the test set for COVID-19 segmentation with U-Net, which uses the best validation models. A comparison of the results of the CT-scan and slice strategies on the test set reveals that the latter presents better results. The differences between the Dice and Jaccard values are statistically significant: t-value = 3.93, *p* < 0.01 (*p* = 0.00033), 95% CI: CT scan—[66.823–79.197], Slice—[82.161–87.159] and t-value = 4.02, *p* < 0.01 (*p* = 0.000308), 95% CI: CT scan—[75.121–82.699], Slice—[84.885–88.535], respectively. Nevertheless, the differences between the accuracy and global accuracy values are not statistically significant: t-value = 0.98, *p* > 0.01 (*p* = 1.67 × 10^−1^), 95% CI: CT scan—[85.031–92.649], Slice—[89.003–92.477] and t-value = 0.67, *p* > 0.01 (*p* = 2.56 × 10^−1^), 95% CI: CT scan—[99.289–99.891], Slice—[99.538–99.842], respectively. To ensure the validity of the t-Student test, we applied the Shapiro–Wilk test to the accuracies, global accuracies, and Dice and Jaccard values, and obtained W(27) = 0.88, *p* < 0.01 (*p* = 7 × 10^−3^); W(27) = 0.77, *p* < 0.01 (*p* = 7.61 × 10^−5^); W(27) = 0.87, *p* < 0.01 (*p* = 5 × 10^−3^); and W(27) = 0.90, *p* < 0.05 (*p* = 2.3 × 10^−2^), respectively.

#### 3.2.2. COVID-19 Segmentation Results with Manual Lung Segmentation

The results for COVID-19 segmentation via the CT-scan and slice strategies that are shown in Table 6 are similar to those shown in Table 5: the Dice and Jaccard values are statistically significant, whereas the accuracy and global accuracy values are not statistically significant. For accuracy, the t-value = 0.37, *p* > 0.01 (*p* = 0.355785), 95% CI: CT-scan—[86.792–94.828], Slice—[89.800–93.320]; for global accuracy, the t-value = 0.54, *p* > 0.01 (*p* = 2.96 × 10^−1^), p5% CI: CT-scan—[97.559–101.741], Slice—[98.426–101.014]; for Dice, the t-value = 3.43, *p* < 0.01 (*p* = 1.13 × 10^−3^), 95% CI: CT-scan—[67.976–80.964], Slice—[82.606–88.094]; and for Jaccard, the t-value = 3.49, *p* < 0.01 (*p* = 9.81 × 10^−4^). To ensure the validity of the t-Student test, we applied the Shapiro–Wilk test to the accuracies, global accuracies, and Dice and Jaccard values, and obtained W(27) = 0.92, *p* < 0.05 (*p* = 0.04); W(27) = 0.81, *p* < 0.01 (*p* = 2.80 × 10^−4^); W(27) = 0.89, *p* < 0.05 (*p* = 1.3 × 10^−2^); and W(27) = 0.88, *p* < 0.05 (*p* = 1.1 × 10^−2^), respectively.

For each DP strategy, the metrics shown in Table 6 are slightly better than those shown in Table 5 with automatic lung segmentation. Nevertheless, there are no statistically significant differences for the CT-scan strategy (accuracy t-value = 0.69, *p* > 0.01 (*p* = 0.260415); global accuracy t-value = 0.32, *p* > 0.01 (*p* = 3.75 × 10^−1^); Dice t-value = 0.29, *p* > 0.01 (*p* = 3.84 × 10^−1^); and Jaccard t-value = 0.34, *p* > 0.01 (*p* = 3.71 × 10^−1^)) or for the slice strategy (accuracy t-value = 0.64, *p* > 0.01, (*p* = 2.65 × 10^−1^); global accuracy t-value = 0.25, *p* > 0.01 (*p* = 4.0 × 10^−1^); Dice t-value = 0.35, *p* > 0.01 (*p* = 3.63 × 10^−1^); and Jaccard t-value = 0.40, *p* > 0.01 (*p* = 3.45 × 10^−1^)).

### 3.3. Segmented CT-Scan Area

**Figure 9:**
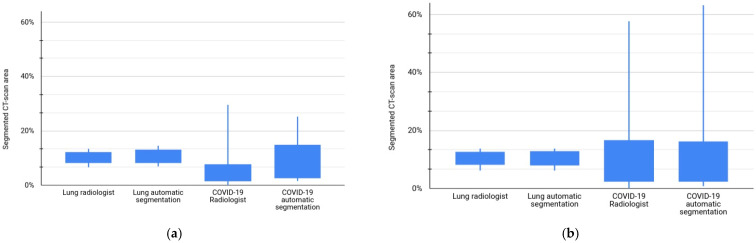
A comparison of the segmented areas of the lungs and COVID-19 patients in test sets, expressed as percentages of the CT-scan area, with the same area segmented by a radiologist: (**a**) CT-scan strategy; (**b**) slice strategy.

The automatically segmented areas of the lungs and COVID-19 patients in test sets, expressed as percentages of the CT-scan area, are compared with the same areas when segmented by radiologists. Figure 9 shows the box graphics for both the lung and COVID-19 segmentations compared with the radiologist segmentations.

As shown in Figure 9, the percentage of area segmented using the slice strategy is very similar to the percentage of area segmented by the radiologists. These results could be explained using Table 5, which compares automatic COVID-19 segmentation results for the CT-scan strategy and the slice strategy. As shown in this table, the metrics for the slice strategy are greater than the metrics for the CT-scan strategy, and the differences are statistically significant.

### 3.4. Comparison of COVID-19 Segmentation in Both Datasets with Interobserver Agreement

We compare the results of the best models for COVID-19 segmentation using the slice strategy with those of interobserver agreement. For this comparison, the 7 CT scans common to Dataset 1 and Dataset 2 were used.

As shown in Table 7, the automatic method using the slice strategy presented better metric values than interobserver agreement did in both datasets. Nevertheless, the differences between the results of the automatic method in both the datasets and interobserver agreement were not statistically significant. In this case, the Shapiro–Wilk test showed that Student’s *t*-test could not be applied. We therefore applied the Mann–Whitney test. For Dataset 1 × interobserver, we obtained the following results: accuracy: U = 16, *p* > 0.01 (*p* = 1.54 × 10^−1^); global accuracy: U = 20, *p* > 0.01 (*p* = 3.05 × 10^−1^); Dice: U = 13, *p* < 0.01 (*p* = 7.93 × 10^−2^); Jaccard: U = 13, *p* > 0.01 (*p* = 7.93 × 10^−2^). For Dataset 2 × interobserver, we obtained the following results: accuracy: U = 6, *p* > 0.01 (*p* = 1.07 × 10^−2^); global accuracy: U = 24.5, *p* > 0.01 (*p* = 0.47608); Dice: U = 17, *p* > 0.01 (*p* = 1.87 × 10^−1^); Jaccard: U = 13, *p* > 0.01 (*p* = 7.92 × 10^−2^).

### 3.5. Lung and COVID-19 Segmentation Examples

**Figure 10:**
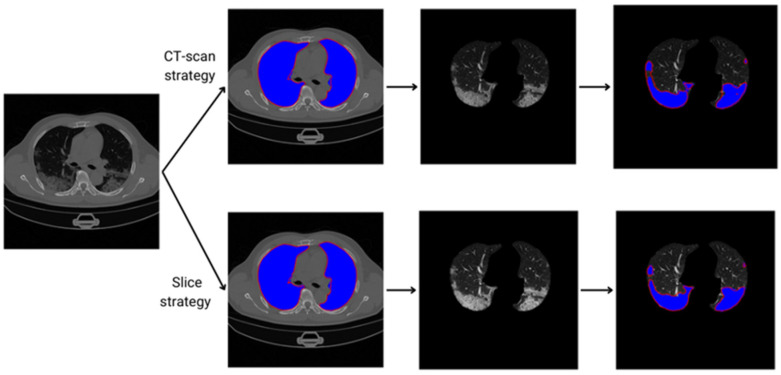
The first row shows the lung and COVID-19 images segmented with U-Net trained with the CT scan strategy. The second row shows the lung and COVID-19 images segmented with U-Net trained with the slice strategy. Radiologist segmentation (Dataset 1) is shown in blue, whereas automatic segmentation is shown in red.

**Figure 11:**
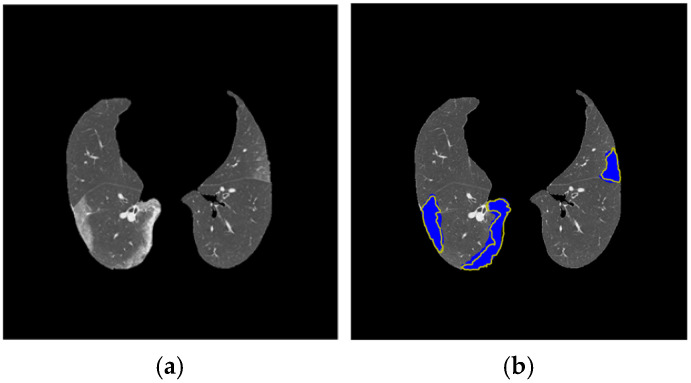
(**a**) Original image; (**b**) agreement between radiologists. Radiologists’ segmentation in Dataset 1 is shown in blue, whereas radiologists’ segmentation in Dataset 2 is shown in yellow.

Figure 10 shows an example of lung and COVID-19 segmentation for a slice of a CT scan using a U-Net trained with a CT-scan strategy and a slice strategy. In the slice shown, there is a great coincidence between the areas segmented with the two strategies. Figure 11 shows an example of COVID-19 segmentation by both groups of radiologists for a slice of a CT scan. As shown, there is a small difference between the two segmentations.

**Figure 12:**
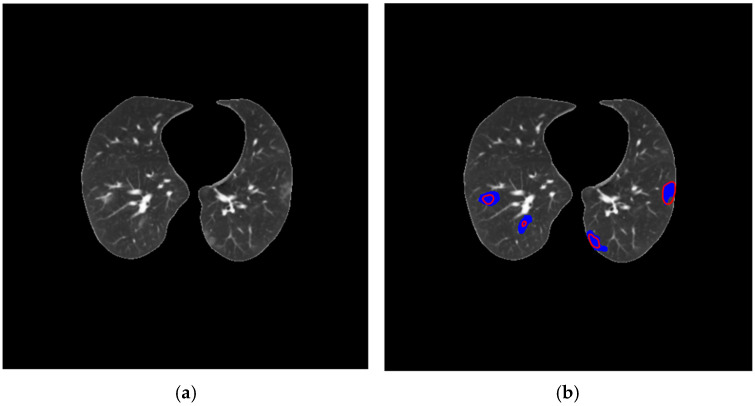
(**a**) Original image with low contrast; (**b**) radiologist segmentation (Dataset 1) is shown in blue, whereas automatic segmentation is shown in red.

In low-contrast areas in CT images, segmentation variations may occur, as shown in Figure 12. In these cases, the difficulty of distinguishing areas with similar pixel intensities compromises the identification of areas affected by COVID-19, as also identified by authors who used one-step segmentation and a CT scan strategy [5,6], one-step segmentation and a slice strategy [17], two-step segmentation and a CT strategy [20], and two-step segmentation and a slice strategy [22].

### 3.6. Computational Cost

The time required to evaluate an image of 512 × 512 pixels is approximately 0.08 s for the CT-scan strategy and 0.07 s for the slice strategy. For an evaluation of a CT scan with 200 images, for example, this implies a time of 16 s using the CT-scan strategy and a time of 14 s using the slice strategy.

## 4. Discussion

From Table 2, which is the result of a literature review on the topic of two-step segmentation, we also observe that better Dice metric values are obtained with the slice strategy than with the CT-scan strategy. Table 5 and Table 6 show that the results obtained in this study with two-step segmentation, with both DP strategies, are competitive with results previously obtained in the literature, which are shown in Table 2.

For example, for COVID-19 segmentation with the CT-scan strategy, the best values for the Dice metric obtained in the literature for two-step segmentation were 71.3% and 67.3% for [20,21], respectively. In this work, with automatic lung segmentation, a Dice value of 73.01% was achieved. On the other hand, for COVID-19 segmentation with the slice strategy, the best values for the Dice metric obtained in the literature for two-step segmentation were 85.0% and 79.8% at [23,24], respectively. In this work, with automatic lung segmentation, a Dice value of 84.66% was achieved.

With respect to lung segmentation, the results obtained in this work are also state of the art. The best value in terms of the Dice metric obtained for the literature on lung and two-step segmentation was 97.18% [25], which was obtained with the slice strategy. Using the CT-scan strategy, the values were 87.90% for the right lung and 85.80% for the left lung in [21]. In this work, we obtained Dice values of 95.29% with the CT-scan strategy and 98.29% with the slice strategy.

U-Net networks were also used in several other previous studies on the segmentation of COVID-19 areas: [8,9,11,13,17]. In this study, although the datasets used are small, we credit the good results obtained to the benefit of combining a U-Net architecture with the following techniques to improve generalization: dropout, L_2_ regularization, and data augmentation. Several combinations of these three techniques were used to obtain the best results.

In this study, we compared the performance of the deep models proposed for COVID-19 segmentation, where a slice strategy was used, with the results obtained using interobserver agreement. In the literature, to the best of our knowledge, only one study [9] has compared COVID-19 segmentation with automatic methods and interobserver agreement. For the CT-scan strategy, the authors obtained Dice values for the COVID-19-infected region segmentation results of the automatic method and those of two experienced radiologists of 0.74 ± 0.28 and 0.76 ± 0.29, respectively, which were close to the interobserver agreement, i.e., 0.79 ± 0.25.

In this work, using a slice-strategy, as shown in Table 7, we obtained Dice values of 83.21 ± 7.52 and 80.78 ± 9.45 for the COVID-19-infected region segmentations of the automatic method and the two groups of radiologists. These were Dataset 1 and Dataset 2, respectively. For interobserver agreement, we obtained a Dice value of 77.69 ± 7.53. The differences between the Dice values of the automatic segmentation method in both datasets and the interobserver agreement method, however, were not statistically significant.

The Dice values of the automatic methods obtained in this work using the slice strategy are higher than the values obtained in [9] with the CT scan strategy. The interobserver Dice value obtained in this work is close to that obtained in [9]. Nevertheless, the standard deviation is greater.

Owing to the small number of common images in the two datasets, it was not possible to make a comparison via the CT-scan strategy. In future works, we intend to use larger datasets, evaluated by two groups of radiologists, to make this comparison possible.

A comparison of the results presented in Table 6 revealed that, for the detection of COVID-19 with two-step segmentation, when the U-Net network and manual lung segmentation were used, the values obtained for COVID-19 segmentation were better for the slice strategy than for the CT-scan strategy in terms of Dice and Jaccard metrics, with a statistically significant difference at the 1% level.

An evaluation of the results presented in Table 5 and Table 6 revealed that, for the same metrics mentioned above, there were no statistically significant differences at the 1% significance level for the accuracy, global accuracy, and Dice and Jaccard metrics when manual or automatic segmentation of the lungs was used.

The COVID-19 pandemic has catalyzed the development of numerous diagnostic tools, including the algorithm presented in this study, which was initially designed for early detection and the risk stratification of patients. Despite overcoming the critical phase of the pandemic, the relevance of the proposed algorithm transcends the context of COVID-19, demonstrating potential application in the identification and quantification of pulmonary alterations with similar radiological characteristics (ground-glass opacities and pulmonary consolidations), often present in conditions such as pulmonary infarction, viral pneumonias, and hypersensitivity pneumonitis.

The segmentation of these areas also allows the quantification of lung involvement, providing an objective parameter for monitoring disease progression and responses to treatment. In scenarios of high demand for imaging services and overloaded health systems, the algorithm can be incorporated into screening systems, optimizing workflow and prioritizing the analysis of exams with suspected significant alterations.

We believe that the algorithm’s ability to segment and quantify areas of lung alteration, with statistically non-significant differences when compared to the segmentation of two groups of radiologists, makes it a valuable tool with an impact beyond the context of COVID-19, contributing to improving the diagnosis and management of different lung diseases.

## 5. Conclusions

The main conclusions of this study are as follows:

1. Using U-NET, COVID-19 segmentation was slightly better for the slice strategy than for the CT-scan strategy. For Dice and Jaccard metrics, there was a difference statistically significant at the 1% significance level.
2. A comparison of the performance of the automatic COVID-19 segmentation results obtained with U-NET and the interobserver agreement in a group of 7 CT scans using the slice strategy revealed no statistically significant differences.
3. When U-Net was used, there were no statistically significant differences in terms of accuracy, global accuracy, and Dice and Jaccard metrics when manual or automatic segmentation of the lungs was performed.

## Figures and Tables

**Table 1:** Summary of published papers on COVID-19 segmentation in CT images via one-step segmentation methods.

Strategy	Ref.	Classifier	Dataset	Train	Validation	Test	Dice (%)
CT scan	[5]	Attention-RefNet	Proprietary	149 CT scans	29 CT scans	32 CT scans	92.75
			COVID-19 CT Seg	3-fold CV	1-fold CV	1-fold CV	91.37
			MICCAI	3-fold CV	1-fold CV	1-fold CV	81.22
	[6]	VB-Net	Proprietary	249 CT scans	-	300 CT scans	91.60 ± 10.00
	[7]	COPLE-Net	Proprietary	378 CT scans	50 CT scans	130 CT scans	80.29 ± 11.14
	[8]	U-Net	Harbin	4-fold CV		1-fold CV	78.30
	[9]	U-Net with backbone ResNet 34	Proprietary	531 CT scans	-	30 CT scans	Radiologist with 20 years’ experience 74.00 ± 0.28. Radiologist with 15 years’ experience 76.00 ± 0.29.
	[10]	nCoVSegNet	LIDC-IDRI	875 CT scans	$\frac{1}{4}$	-	-
			MosMedData	40 CT scans	-	10 CT scans	68.43
			Coronacases	-	-	10 CT scans	68.94
	[11]	U-Net	Proprietary	50 CT scans		130 CT scans	67.00
Slice	[12]	CNN	COVID-CT	70%	10%	20%	92.00
	[13]	Based on U-Net	COVID-CT	1069 slices	150 slices	150 slices	88.00
			COVID-19 CT				
			Henri Becquerel Cancer Center				
	[14]	O-Net	COVID-19 CT Seg	70%	-	30%	86.00
			MosMedData				
	[15]	QC-HC U-Net	MSD	80%	-	20%	85.31
			COVID-19 CT Seg	80%	-	20%	
	[16]	STCNet	CC-CCII segmentation	70%	-	30%	82.78
			COVID-19 CT Seg	70%	-	30%	79.92
	[17]	JSC	COVID-CS	2794 slices	-	1061 slices	78.50
	[18]	Semi-Inf-Net	COVID-19 CT	45 slices	5 slices	50 slices	73.90
			COVID-19 CT collection	1600 slices	-	-	
	[19]	SSInf-Net	COVID-19	698 slices	114 slices	117 slices	63.00
			ICTCF	6654 slices	-	-	

**Table 2:** Summary of published papers on COVID-19 segmentation in CT images via two-step segmentation methods.

Strategy	Ref.	Classifier	Dataset	Train	Valid.	Test	Dice (%)
CT scan	[20]	Lungs Model by another author COVID-19 3D dense CNN	Proprietary	64 CT scans	23 CT scans	77 CT scans	COVID-19 Institute 1 71.30 Institute 2 65.70 Institute 3 64.10
				58 CT scans	14 CT scans	87 CT scans	
	[21]	Lungs nnU-Net COVID-19 nnU-Net	COVID-19 CT Seg	4-fold CV with 16 CT scans	-	1-fold CV with 4 CT scans	Right lung 87.90 ± 9.30 Left lung 85.80 ± 10.50 COVID-19 67.30 ± 22.30
			MosMed	-	-	50 CT scans	COVID-19 58.80 ± 20.60
	[22]	Lungs Model by another author COVID-19 U-Net	Proprietary	COVID-19 Infection 80 CT scans	COVID-19 Infection 7 CT scans	COVID-19 Infection 62 CT scans	COVID-19 Infection 63.20
			Proprietary	COVID-19 Consolidation 19 CT scans	-	-	COVID-19 Consolidation 62.80
			MedSeg	-	-	COVID-19 Consolidation 9 CT scans	
Slice	[23]	Lungs Model by another author COVID-19 U-Net	COVID-19 CT	4-fold CV with 80 slices	-	1-fold CV with 20 slices	COVID-19 85.00
	[24]	Lungs Model by another author COVID-19 Anam-Net	COVID-19 CT Seg (exp.2)	3-fold CV	-	1-fold CV with 545 slices	COVID-19 79.80
			COVID-19 CT	270 slices	-	-	COVID-19 75.50
			MedSeg	-	-	704 slices	
			-	Model trained in exp. 2	-	-	COVID-19 66.40
			MedSeg	-	-	704 slices	
	[25]	Lungs RCNN-ResNetSt-200 COVID-19 RCNN-ResNetSt-200	NSCL	50,756 slices	-	1222 slices	Lungs 97.18
			COVID-19 CT Seg				
			MosMed	5854 slices	-	1117 slices	COVID-19 78.06
			MSD				
			COVID-19 CT Seg				
	[26]	Lungs Model by another author COVID-19 SegNet	COVID-19 CT	72 slices	10 slices	18 slices	COVID-19 74.90

**Table 3:** Hyperparameters used for classifier training.

Hyperparameter	Value
Optimizer	ADAM
Batch size	32
Training stop method	Early stop
Initial learning rate	10^−3^
Reduction learning rate factor	10^−1^
Interval between learning rate reductions	20 epochs
Minimum learning rate	10^−45^
Maximum number of training epochs	600
Loss function	Binary cross entropy
Dropout rate	0.5
L_2_ regularization factor	10^−3^
Data augmentation	10 rotations of 36°

**Table 4:** Results of lung segmentate on in the test set via a U-Net network.

Strategy	Acc. (%)	Global Acc. (%)	Dice (%)	Jaccard (%)
CT scan	97.60 ± 2.00	99.01 ± 1.34	95.29 ± 5.21	95.15 ± 5.12
Slice	98.76 ± 0.76	99.66 ± 0.19	98.29 ± 0.83	98.13 ± 0.89

**Table 5:** Results of COVID-19 segmentation in the test set obtained with automatic lung segmentation and the U-Net method.

Strategy	Acc. (%)	Global Acc. (%)	Dice (%)	Jaccard (%)
CT scan	88.84 ± 5.83	99.59 ± 0.46	73.01 ± 9.47	78.91 ± 5.80
Slice	90.74 ± 3.76	99.69 ± 0.33	84.66 ± 5.41	86.71 ± 3.95

**Table 6:** Results of COVID-19 segmentation in the test set obtained with manual lung segmentation and using the U-Net network.

Strategy	Acc. (%)	Global Acc. (%)	Dice (%)	Jaccard (%)
CT-scan	90.81 ± 6.15	99.65 ± 0.32	74.47 ± 9.94	79.90 ± 6.04
Slice	91.56 ± 3.81	99.72 ± 0.28	85.35 ± 5.41	87.29 ± 4.36

**Table 7:** A comparison between the COVID-19 segmentation of the proposed method using the slice approach with the results of interobserver agreement.

Method	Acc. (%)	Global Acc. (%)	Dice (%)	Jaccard (%)
Automatic method in Dataset 1	89.64 ± 4.68	99.51 ± 0.43	83.21 ± 7.52	85.67 ± 5.45
Automatic method in Dataset 2	99.44 ± 3.3	99.50 ± 0.04	79.30 ± 7.90	82.90 ± 6.54
Interobserver agreement	88.08 ± 5.16	99.52 ± 0.43	77.69 ± 7.53	81.76 ± 4.89

## Data Availability

The datasets are available in [27,28].
